# Combining Molecular Dynamics Simulations and Biophysical Characterization to Investigate Protein-Specific Excipient Effects on Reteplase during Freeze Drying

**DOI:** 10.3390/pharmaceutics15071854

**Published:** 2023-06-30

**Authors:** Suk Kyu Ko, Gabriella Björkengren, Carolin Berner, Gerhard Winter, Pernille Harris, Günther H. J. Peters

**Affiliations:** 1 Department of Chemistry, Technical University of Denmark, 2800 Kongens Lyngby, Denmark; mr.kyu1229@gmail.com (S.K.K.); gabriella.bjorkengren@gmail.com (G.B.); 2 Department of Pharmacy, Ludwig Maximilian University of Munich, 81377 Munich, Germany; carolin.berner@cup.uni-muenchen.de (C.B.); gerhard.winter@lrz.uni-muenchen.de (G.W.); 3 Department of Chemistry, University of Copenhagen, 2100 Copenhagen, Denmark; phharris@chem.ku.dk

**Keywords:** freeze drying, molecular dynamics, coarse-grained simulations, Reteplase, formulation development, protein stability, arginine

## Abstract

We performed molecular dynamics simulations of Reteplase in the presence of different excipients to study the stabilizing mechanisms and to identify the role of excipients during freeze drying. To simulate the freeze-drying process, we divided the process into five distinct steps: (i) protein–excipient formulations at room temperature, (ii) the ice-growth process, (iii)–(iv) the partially solvated and fully dried formulations, and (v) the reconstitution. Furthermore, coarse-grained (CG) simulations were employed to explore the protein-aggregation process in the presence of arginine. By using a coarse-grained representation, we could observe the collective behavior and interactions between protein molecules during the aggregation process. The CG simulations revealed that the presence of arginine prevented intermolecular interactions of the catalytic domain of Reteplase, thus reducing the aggregation propensity. This suggests that arginine played a stabilizing role by interacting with protein-specific regions. From the freeze-drying simulations, we could identify several protein-specific events: (i) collapse of the domain structure, (ii) recovery of the drying-induced damages during reconstitution, and (iii) stabilization of the local aggregation-prone region via direct interactions with excipients. Complementary to the simulations, we employed nanoDSF, size-exclusion chromatography, and CD spectroscopy to investigate the effect of the freeze-drying process on the protein structure and stability.

## 1. Introduction

Protein therapeutics have been considered one of the most widely applied forms of drugs. However, proteins in a liquid formulation exhibit several chemical and physical instabilities. Therefore, the stability and shelf-life of some protein-based drugs are usually improved by solidifying the drug-containing formulations. The lyophilization (freeze-drying) technique is usually applied to achieve the solidification of the desired protein-based drug products [1]. However, the lyophilization process involves freezing and drying of the formulations and both processes can cause instabilities in the processed protein drugs. During freezing, the formation of ice can induce a local increase of the solute concentrations, pH shifts, and protein adsorption at the ice–water interface. The removal of water molecules around the protein during the drying process results in a loss of solvent–protein hydrogen bonds and a decrease of the hydrophobic effect that can cause the unfolding of the protein. Therefore, freeze-dried formulations require different excipients to provide cryo- and lyo-protection [2]. Recently, Arsiccio et al. [3,4,5,6,7,8,9,10,11] performed a series of extensive investigations on the computational modeling of the protein–excipient systems during freeze-drying conditions. The authors performed conventional molecular dynamics (MD) simulations and biased simulations to study the effect of common excipients on protein stability during the freeze-drying process [3,4,5,7] and the effect on an ice–water interface [8,10,11], respectively. The studies revealed that it was possible to simulate the effect of preferential exclusion and water replacement [4], which could be considered a universal protein-unspecific protection mechanism of excipients. In this study, we focused on Reteplase to investigate the stabilization mechanism that was protein specific that was usually caused by direct interactions between protein and excipients; we also studied the effects of excipients as a protein-stabilizing agent. Reteplase is a recombinantly produced tissue plasminogen activator that is commercially available as a freeze-dried product (commercial name: Retavase^®^) and used in the treatment of acute myocardial infarction [12,13]. Reteplase is highly unstable without arginine (ARG) or tranexamic acid (TXA). It is known that the solubility of Reteplase can be highly improved by adding ARG [14]. The ARG-containing Reteplase formulation has been studied by Tischer et al. [14] using fluorescence spectroscopy. Between 100 and 200 mM of ARG Reteplase remained stable with no change in the unfolding profile. However, it was possible to observe an instant aggregation of Reteplase in the absence of ARG; the aggregation could be not prevented by adding NaCl [14]. Interestingly, it has also been suggested that TXA could be used to prevent the aggregation of Reteplase. Mousavi et al. documented the anti-aggregation effect of TXA on the Reteplase formulations [15]. The authors suggested that the TXA could stabilize Reteplase by binding to the kringle-2 domain without disrupting the native conformation. Furthermore, the binding spots of kringle-2 domains have been suggested in the literature. Mutagenesis and molecular docking studies have proposed Lys40 as one of the first binding spots [16]. In addition, the second interacting region could be driven by hydrophobic interactions between the aliphatic group of the ligand and the hydrophobic region of the kringle-2 domain [17].

This study aimed to study the stabilization of ARG and TXA during the freeze-drying process; our main focus was the Reteplase specific protein stabilization mechanism that involved interaction between protein and excipients [18]. We included sucrose (SUC) as a protein unspecific stabilizer model. Since the instability of Reteplase can be observed as an aggregation, coarse-grained (CG) simulations were used to study protein–protein interactions (PPIs) between Reteplase monomers with and without the presence of the excipient. Complementarily, we characterized Reteplase in the presence and absence of the excipients with various biophysical techniques. Samples undergoing freeze-thaw cycles and lyophilization were analyzed using differential scanning fluorimetry, circular dichroism spectroscopy, and high-performance size exclusion chromatography to assess the protein stability under different conditions and to compare with computational results.

## 2. Materials and Methods

### 2.1. Structure

**Figure 1:**
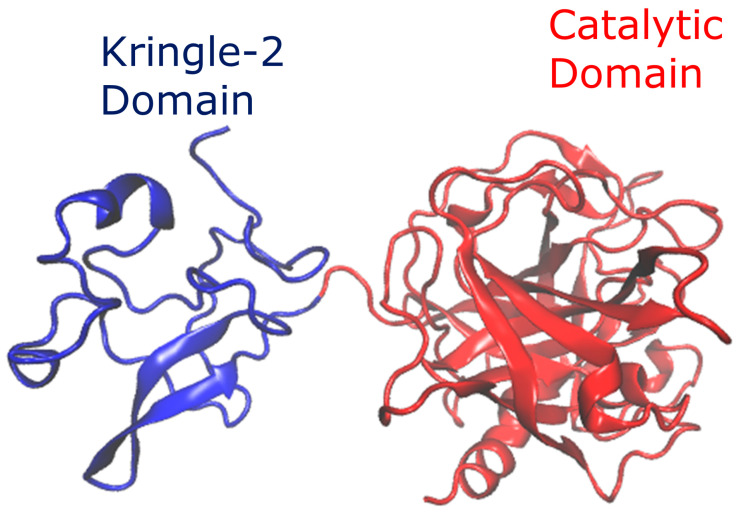
Chimeric model of Reteplase. The structures were created by combining 1TPK and 1BDA to model the kringle-2 domain and catalytic domain, respectively. Modeller software was used to build the structure.

Reteplase contains 355 residues; it is a multi-domain protein that contains the kringle-2 domain and the catalytic domain. Reteplase is a recombinant protein with a longer half-life than its native protein [13,19]. The structure of Reteplase can be characterized as a β-sheet and loop-rich protein (Figure 1). The complete structure of Reteplase has not been solved in the literature. However, the kringle-2 domain (1PK2 [20] and 1TPK [21]) and catalytic domain (1BDA [22]) have been solved. Modeller software version 9.21 [23] was applied to build the complete structure of Reteplase as a chimeric structure, with 1TPK and 1BDA as the templates.

### 2.2. Coarse-Grained Simulations

Gromacs 2018 [24] was used to run the CG simulations. The CG model was described and parameterized with the SIRAH force field [25,26,27]. PDB2PQR [28] was used to create the PQR structures, which were used as an input file to generate the CG structures. While generating the PQR structures, the protonated state of residue was kept in a standard format to model Reteplase at physiological conditions. After the generation of the PQR structure, the input PQR files were coarse-grained using the SIRAH toolbox [26]. After the coarse graining, the CG monomer structures were duplicated in x-, y-, and z directions. Eight monomers were generated in the system and the center of the mass distance between monomers was 85 Å in x, y, and z directions. The protein edge to the simulation box edge distance was defined as 7.5 Å. The protein concentration of the simulations could be controlled by defining the size of the simulation box with a limited number of protein monomers. If the protein concentration was too high, then the monomers in the simulations would immediately aggregate. The biggest challenge of simulating the protein in a realistic concentration was the limitation of the computational power, since a large amount of water molecules was needed to obtain a realistic protein concentration scale. As conducted in our previous study [29], the sizes of the simulation boxes were adjusted empirically to achieve an efficient sampling of the PPIs. Each system was solvated with SIRAH CG water molecules, WT4 [30]. After solvation, the system was neutralized by adding ions. The system was divided into two different conditions: the system with 10% *w*/*w* ARG and the system without any excipients. We considered ARG as the CG excipient model, since ARG is a widely used amino acid excipient to obtain stable protein formulations [31]. After the generation of the CG systems, the systems were subjected to minimization using 50,000 maximum steps of steepest descent and conjugated gradient algorithm. A cutoff distance of 12 Å was used to reduce the amount of the non-bonding interactions. We performed an additional relaxation to the ARG-containing system by running 10 K NVT ensemble simulations for 10 ns. The heating was performed with the Berendsen thermostat and barostat [32]. The system was heated to 300 K at a pressure of 1 bar. In order to obtain an NPT ensemble, we coupled the system to the stochastic velocity rescaling thermostat [33] and the Parrinello–Rahman barostat [34]. The systems were equilibrated for 500 ns. The final production run was performed for 3 µs. The time step size was set to 10 fs. For each condition, the simulations were performed in triplicate; the initial atom velocities were different for every simulation.

The aggregation propensity of the proteins was analyzed by determining the number of PPIs observed in the simulations. A monomer–monomer interaction was counted when the distances between one residue pair between two different Reteplase monomers had a distance less than 4 Å. The determined PPIs were normalized to the condition with the weakest observed PPIs (Reteplase + 10% *w*/*w* ARG).

Additionally, we generated heatmaps (Figures S1 and S2) to identify the residues that were involved in the PPIs. Each heatmap pixel represented the number of PPIs of the corresponding interaction pair. The color code of the pixel was scaled to the strongest interacting residue pair at the non-aggregating condition (Reteplase + ARG). If the interactions were occurring less than 25% of the strongest interacting residue pair at Reteplase + ARG, then the interactions were removed in the heatmap. The residues that participated in the PPIs are investigated to obtain an overview of the PPI profile of Reteplase.

### 2.3. Freeze-Drying Simulations

**Figure 2:**
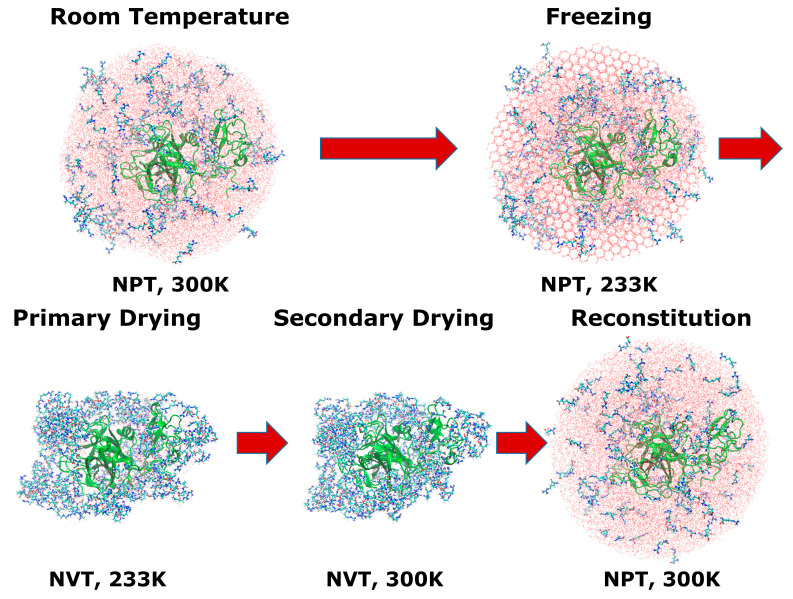
The full cycle of the developed FD simulation protocol. The liquid and frozen conditions were simulated in the NPT ensemble. The drying process was simulated in the NVT ensemble. The arrows indicate that the final frame of the previous simulations was used to initiate the next simulations.

We studied a complete cycle of the freeze-drying (FD) process using MD. After simulating the full cycle of the FD process, we re-added water back to the system to simulate the reconstitution behavior of proteins. The simulation steps in the developed protocol [18] were inspired by the MD investigation performed by Arsiccio et al. [4,9] and Urbassek et al. [35]. We used AMBER 20 software [36] to perform the simulations. Figure 2 describes an overview of the full-cycle FD simulations.

The FD simulations were executed in five different steps. The simulations were performed in chronological order: (1) simulations of the liquid formulations at room temperature, (2) freezing simulations with freezing-inducing ice boxes, (3) first drying simulations, (4) secondary drying simulations, and (5) re-addition of water to simulate the reconstitution of the system. Each FD simulation step was carried out for 400 ns, resulting in a total FD simulation time of 2 µs. The Langevin thermostat [37] and the Monte Carlo barostat [38] were used to control the temperature and pressure, respectively. The Langevin thermostat was coupled to the system with a collision frequency of 5 ps^−1^. The non-bonding interactions were calculated with a cutoff distance of 12 Å. The long-range interactions were calculated using the particle mesh Ewald method [39]. The SHAKE algorithm [40,41] was used to remove the vibration of the hydrogen bonds. 

The FF14sb [42] forcefield was used to parameterize Reteplase. We obtained the structure of ARG, TXA, and SUC from the ZINC database [43]. In the early stage, we used the GAFF2 AMBER force field [44] to describe the excipients. We experienced that the GAFF2-based excipient had a strong binding affinity towards the protein; this result has been also reported in the literature [45]. To obtain more accurate protein–excipient interactions, the non-bonding parameter of SUC was parameterized with Kirkwood–Buff derived CMARHMM36 parameters, which was specifically designed to reproduce preferential exclusion of sugar molecules [46]. We used the CHARMM36 protein parameter to [47] describe the non-bonding parameter of ARG; TXA was re-parameterized with the non-bonding parameter of the CHARMM-based ligand builder [48]. An overview of the simulated system is shown in Table 1. We used a concentration range of 10 to 100 mM excipients to mimic the experimental conditions. In addition, a 10% *w*/*w* excipient concentration was used to enhance the effect of excipients on protein structure and stability.

The starting system of the FD simulations was defined in a cubic box, with a protein-box edge distance of 15 Å. The water molecules in the system were described with the TIP4P/Ewald model [49]. After the addition of the water and excipients, we neutralized the system by adding ions (Table 1). The initial system was subjected to minimization. The first 5000 minimization steps were carried out in the steepest descent algorithm and the remaining 5000 steps were computed using the conjugate gradient algorithm. The system was heated to 300 K in an NVT ensemble for 0.3 ns. The equilibration run was performed for 2.2 ns in an NPT ensemble with a pressure of 1 bar. The production run of the room temperature simulations was carried out for 400 ns. The last frame of the simulations was extracted as a PDB file. The extracted PDB file was used as an input file to set up the freezing simulations. 

To describe the ice formation of the system, we used TIP4P/ICE [50] to model the ice. The freezing was induced by covering the xyz surfaces of the final frame of the room temperature simulations with three hexagonal ice blocks. We used GenIce software version 0.22.11 [51] to create the ice blocks. A short minimization was performed to relax the bad contact between the ice and the system. We used 500 steps of steepest descent and conjugated gradient algorithms to minimize the system. The heating was conducted in two steps: (1) NVT simulations at 100 K for 0.3 ns and (2) NPT simulations at 233 K for 0.3 ns. The equilibration and production runs were performed in a similar scheme to the room temperature simulations, but the temperature was kept to 233 K. To mimic the primary drying condition, the water molecules that were 3 Å away from the protein were removed from the final frame of the freezing simulations. 

The vacuum condition of the primary drying process was created by defining an NVT condition with the box edge to protein distance of 50 Å. The water molecules were parameterized with TIP4P/ICE to mimic the sublimation condition. The system was heated for 0.3 ns; after heating, the production run was carried out for 400 ns. The final frame of the production run was extracted as a PDB file. Most of the water molecules were removed from the system. However, the residual water molecules were modeled by preserving the water molecules that were placed similarly to the crystal water that could be found in the original X-ray structure files. The output of the primary drying was used as an input to start the secondary drying simulations.

During the secondary drying simulations, the residual waters were parameterized with a TIP4P/EW forcefield. The system was heated to 300 K for 0.3 ns. The secondary drying simulations were carried out in vacuum conditions for 400 ns at the temperature of 300 K. 

To start the reconstitution simulations, the dried output from the secondary drying simulations was re-solvated by re-adding the TIP4P/EW water back to the system. The re-solvation occurred in a cubic simulation box with a protein-box distance of 15 Å. A short minimization was carried out to eliminate the bad contacts. The minimization was performed using 500 steps of the steepest descent and the conjugate gradient algorithms. The remaining simulations were carried out in a similar scheme that was used in the room temperature simulations. The output of the FD simulations was analyzed using VMD [52] and a Python script based on the MDTraj library [53]. 

### 2.4. Materials

Reteplase was provided by Roche Diagnostics GmbH (Mannheim, Germany); it was formulated in 250 mM phosphoric acid, pH 7.2, and +500 mM ARG + 0.1% polysorbate 80. The protein concentration was measured spectrophotometrically using a NanoDrop 2000 (Thermo Fisher Scientific, Wilmington, NC, USA). All chemicals were of molecular biology or multicompendial grade and were purchased either from Sigma (St. Louis, MO, USA) or Thermo Fisher Scientific (Dreieich, Germany). All solutions were prepared with ultrapure water from a Sartorius arium^®^ pro system (Sartorius Corporate Administration GmbH, Göttingen, Germany). 

### 2.5. Sample Dialysis and Preparation

The buffer was exchanged by extensive dialysis to 100 mM potassium phosphate buffer, pH 7.2, without and with the respective excipients (10 mM and 100 mM ARG or TXA) for Reteplase for 24 h at 2–8 °C using a Spectra/Por^®^ dialysis membrane (cutoff 6–8 kDa, Spectrum Laboratories, Rancho Dominguez, CA, USA). The samples were collected in microcentrifuge tubes and centrifuged at 10,000× *g* for 10 min. Reteplase samples were diluted to a final concentration of 0.4 g/L. All samples were filtered with 0.02 µm Anotop^®^ membrane filters (Whatman, FP 30/0.2 CA-S, GE Healthcare, Buckinghamshire, UK) into 2R Type I glass vials, closed with rubber stoppers, and crimped. 

### 2.6. Freeze-Thaw Cycles and Lyophilization

The freeze-thaw and freeze-drying runs were performed on a Martin Christ Epsilon 2-6D (Martin Christ, Osterode am Harz, Germany) equipped with a comparative pressure measurement system containing a Pirani pressure gauge and a capacitive manometer. Samples were freeze-thawed in 5 cycles with a freezing rate of −1 °C/min to −50 °C. After 120 min at −50 °C, the samples were thawed at 1 °C/min to 20 °C with a 120 min hold.

Freeze drying was performed as follows: Shelf temperature was decreased from 20 °C to −3 °C, with a ramp rate of −1 °C/min and equilibration for 60 min. Subsequently, the shelf temperature was decreased to −50 °C, with a freezing rate of −0.3 °C/min and equilibration for 120 min. Then, a vacuum of 0.09 mbar was applied and the shelf temperature was increased to −20 °C, using a ramp rate of 0.5 °C/min. This temperature was held for 35 h and then increased to 5 °C, using a ramp rate of 0.1 °C/min. The temperature was further increased to 25 °C, with a ramp of 0.2 °C/min, and held for 7 h. Venting was performed with nitrogen at 800 mbar with subsequent vial closing. 

### 2.7. Differential Scanning Fluorimetry (nanoDSF)

nanoDSF was used to study the thermal unfolding and aggregation of Reteplase with and without the respective excipients. Standard nanoDSF™ grade capillaries were filled with the samples and sealed. The Prometheus NT.48 (NanoTemper Technologies, Munich, Germany) system was used to apply a temperature ramp of 1 °C/min from 20 to 100 °C. The intrinsic protein fluorescence intensities at 330 and 350 nm were measured after excitation at 280 nm. Simultaneously, the aggregation of the samples was monitored by the integrated back reflection detection optics. The apparent protein melting temperatures (*T*_m_) were determined by PR.ThermControl software V2.1 (NanoTemper Technologies, Munich, Germany) from the thermal unfolding curves. The same software was used to determine the aggregation onset temperature (*T*_agg_) from the increase in the signal from the aggregation detection optics.

### 2.8. Circular Dichroism (CD) Spectroscopy

Near-UV circular dichroism spectra of samples before and after freeze/thaw or lyophilization were collected at 25 °C with a Jasco J-810 spectropolarimeter (JASCO Deutschland GmbH, Pfungstadt, Germany). The measurements were performed in a quartz cuvette (Hellma GmbH, Muellheim, Germany), with a 10 mm wavelength path. Three accumulations of each sample were taken at a speed of 20 nm/min. The spectrum of the respective buffer was subtracted for each sample and smoothing of the spectra was performed using the Savitzky–Golay algorithm with 9 smoothing points. 

### 2.9. High-Performance Size Exclusion Chromatography (HP-SEC)

A Dionex Ultimate 3000 system (Thermo Fisher, Dreieich, Germany) was used for the HP-SEC analysis of the Reteplase samples. An amount of 10 µL of each sample was injected in triplicate on a Superdex 200 Increase 10/300 GL column (GE Healthcare Bio-Sciences, Uppsala, Sweden) after centrifugation at 10,000× *g* for 10 min; the elution of the protein was detected by UV spectrometry at 280 nm. The mobile phase consisted of 100 mM sodium phosphate, pH 7.2, and 0.05% NaN_3_. The flow rate was set to 0.5 mL/min. The chromatograms were integrated with Chromeleon V7 (Thermo Fisher, Dreieich, Germany). Monomer recovery was calculated by integration of the peak area and relative comparison of this peak area before and after freeze/thaw and lyophilization. 

## 3. Results and Discussion

The effect of ARG and TXA on Reteplase was investigated by different MD simulation approaches. Reteplase is highly aggregation-prone without the presence of ARG or TXA. To have an overview of the PPI profile of Reteplase, we carried out CG simulations of multiple Reteplase monomers with and without the presence of ARG. The FD-induced unfolding behavior of Reteplase and the stabilization mechanism of the excipient were studied using full atomic MD simulations. The simulation results were verified using experimental data.

### 3.1. Protein–Protein Interaction Study of Reteplase

**Figure 3:**
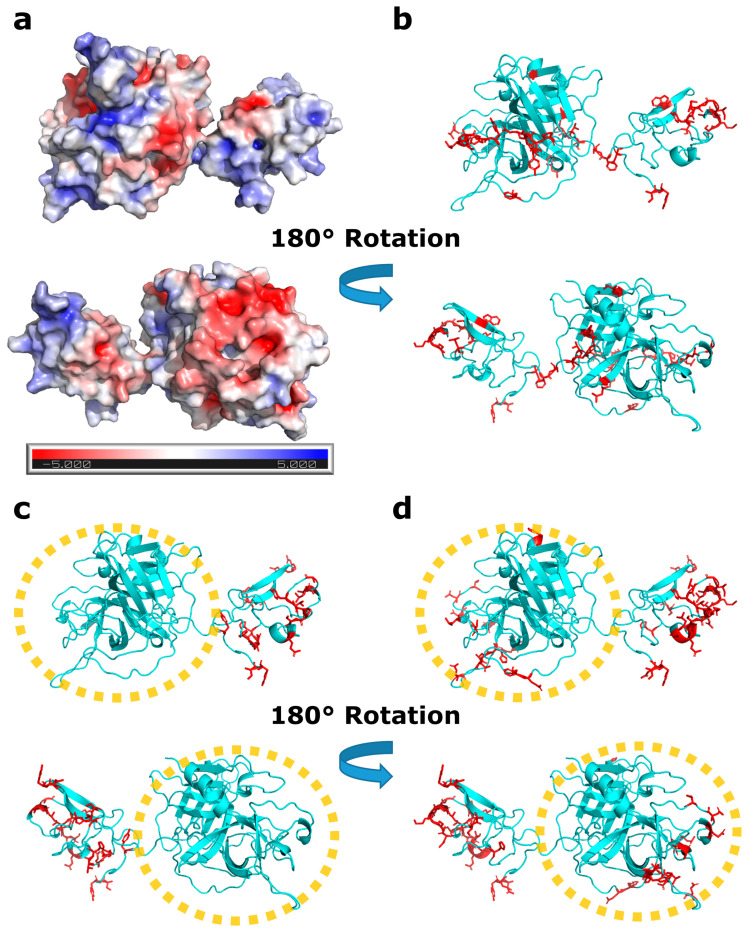
The protein–protein interaction (PPI) profile of Reteplase. (**a**) Electrostatic potential map showing local positively and negatively charged patches. (**b**) Visual representation of the hydrophobic aggregation-prone residues as red sticks. The aggregation-prone residues were detected using the A3D algorithm [56]. (**c**,**d**) Interaction-prone residues observed in the CG simulations. Regions involved in PPIs during the CG simulations are shown as red sticks. (**c**) PPIs hotspots in the presence of ARG 10% *w*/*w*. (**d**) PPI hotspots without excipients. The catalytic domain of Reteplase is marked with an orange circle.

The aggregation behavior of Reteplase was studied using CG simulations of multiple Reteplase monomers and experimentally verified with *T*_m_ and *T*_agg_ measurements, as further discussed below. Reteplase formed aggregates without the addition of ARG [54] or TXA [15]. Both ARG and TXA consisted of positively and negatively charged functional groups. This indicated that the electrostatic properties played a crucial role in the aggregation behavior of Reteplase. We, therefore, calculated the electrostatic potential surface using the APBS [55] and PDB2PQR [28] programs. Reteplase contained a high number of charged residues, which generated both positively and negatively charged local spots on the surface (Figure 3a), which could be the cause of aggregation. To investigate the involvement of hydrophobic interactions, we predicted aggregation-prone residues using the Aggrescan3d (A3D) algorithm [56] (Figure 3b). 

A few aggregation-prone hotspots could be observed in Reteplase (Figure 3b). Reteplase contained both hydrophobic patches and locally charged patches, which implied that aggregation was probably driven by a combination of both electrostatic and hydrophobic forces.

To investigate the effect of ARG on PPIs, we performed CG simulations of Reteplase with 10% *w*/*w* and without ARG. We monitored the aggregation propensity by counting the number of PPIs during the simulations. The residues that were involved in PPI are shown in Figure 3c,d. 

The calculation of the relative number of PPIs in the presence and absence of ARG revealed that the CG simulations without ARG had twice as many PPIs compared with the Reteplase + ARG simulations. Visual inspection of the trajectory showed that Reteplase monomers in the presence of ARG were forming much smaller oligomers than Reteplase without ARG (Figure S3).

The effect of ARG on the CG simulations could be noticed in the catalytic domain (Figure 3c,d). The catalytic domain became much more aggregation-prone when ARG was removed from the system (Figure 3c). The strong interactions in the system containing ARG were mainly observed in the kringle-2 domain (Figure 3d). The CG simulations indicated that the presence of ARG was important to reduce the interaction between Reteplase monomers. Considering that the catalytic domain contained many negative patches (Figure 3a), one may argue that the binding of ARG to the catalytic domain was a protein-specific mechanism involved in preventing aggregation. 

### 3.2. Molecular Dynamics Simulations of the Freeze-Drying Process

**Figure 4:**
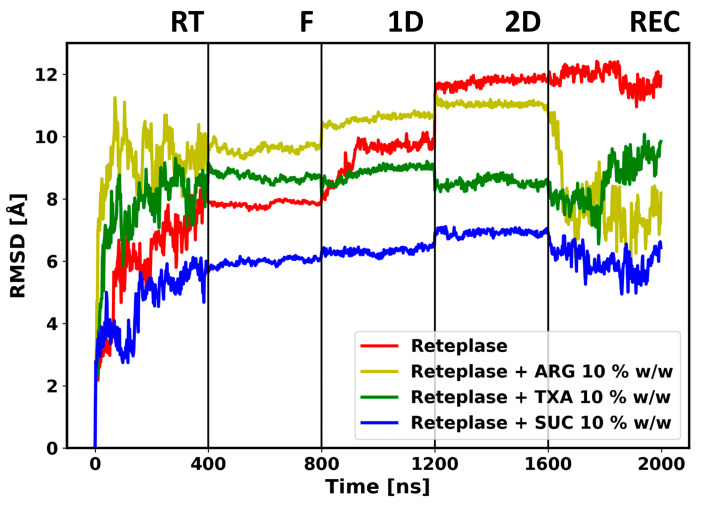
Representative RMSD vs. time plot of Reteplase during the FD simulations. Room temperature (RT), freezing (F), primary drying (1D), secondary drying (2D), and reconstitution (REC) denote the different steps of the FD process, which are separated by vertical lines. The reference state of the RMSD calculation is the starting structure of the simulations.

The FD simulations were carried out with five different consecutive simulation steps. Three excipients were considered in this study: ARG, TXA, and SUC. To obtain an overview of the effect of the FD simulations on the structure of Reteplase, we calculated the time evolution of the root mean square deviation (RMSD) of Reteplase to monitor the global structural changes during the FD process. Representative RMSD as a function of simulation time of Reteplase only, Reteplase + 10% *w*/*w* ARG, and Reteplase + 10% *w*/*w* SUC are shown in Figure 4; the time courses of the RMSD for the individual simulations are provided in Figure S4.

**Figure 5:**
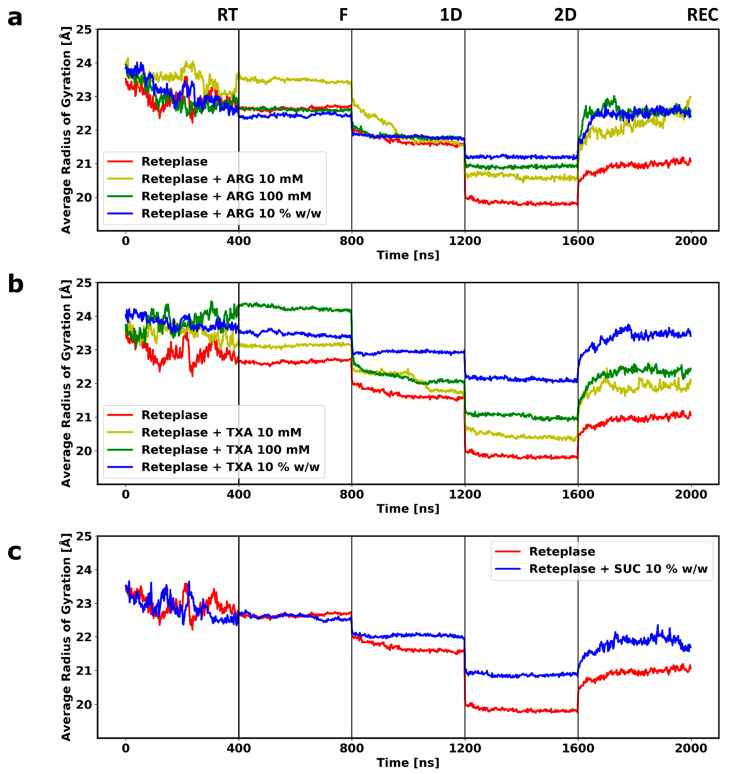
The plot of the protein radius of gyration. Three systems with different excipients at different concentrations are plotted: (**a**) Reteplase with ARG, (**b**) Reteplase with TXA, and (**c**) Reteplase with SUC. Each time course is obtained from the average of two independent simulations. Room temperature (RT), freezing (F), primary drying (1D), secondary drying (2D), and reconstitution (REC) denote the different steps of the FD process, which are separated by vertical lines.

**Figure 6:**
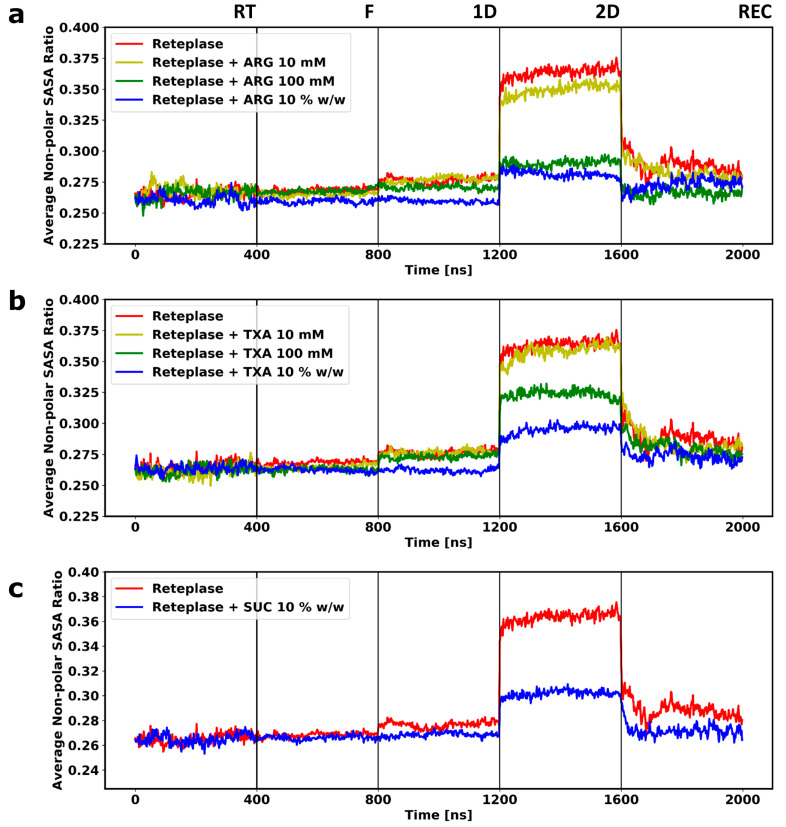
The time course of the ratio between non-polar and total solvent accessible surface area (SASA). Three systems with different excipients at different concentrations are plotted: (**a**) Reteplase with ARG, (**b**) Reteplase with TXA, and (**c**) Reteplase with SUC. Each time course is obtained from the average of two independent simulations. Room temperature (RT), freezing (F), primary drying (1D), secondary drying (2D), and reconstitution (REC) denote the different steps of the FD process, which are separated by vertical lines.

During the simulations at room temperature, high fluctuation could be observed for Reteplase (Figure 4), reflecting a reorientation of the kringle-2 domain with respect to the catalytic domain. This could be seen from the time evolution of the RMSD of the individual domains (RMSD ≈ 2.5 Å) (Figure S5) compared with the combined analysis (RMSD ≈ 8 Å). The inspection of the β-sheet content during the simulations revealed that the overall secondary structure of Reteplase was well preserved at the room temperature simulations (Figure S6). As excepted, the simulations at the FD conditions (from freezing (F) to secondary drying (2D)) caused a reduction in protein fluctuation. Due to the low temperatures during freezing and primary drying, i.e., low thermal energy, the movement of protein was highly restricted at the initial stage of the freeze-drying process. Even though the temperature was increased in the secondary drying stage, the removal of water molecules from the system led to the solidification of the protein. This could be observed by a decrease in the RMSD fluctuation (Figure 4). Notably, the structure of Reteplase changed during the drying and reconstitution stages. This conformational change occurred as a continuous increase or decrease in RMSD. The dried protein structure would be reconfigured when water molecules were added back to the system. However, no large fluctuation could be observed after the reconstitution of Reteplase without any excipients, indicating that the conformational changes occurring during the FD simulations were not reversible. Note that SUC did not cause significant conformational changes during the reconstitution simulations. Since the RT fluctuation of Reteplase was far greater than the fluctuation during the FD process, it may have seemed that RMSD fluctuation was stable during the FD simulations. However, if we only plotted the RMSD changes during the FD process, the RMSD time series showed a continuous change during the FD and reconstitution process (Figure S7). The conformational changes of Reteplase were further inspected by plotting the radius of gyration (Figure 5) and the ratio between non-polar and total solvent-accessible surface area (SASA) (Figure 6) during the FD simulations.

**Figure 7:**
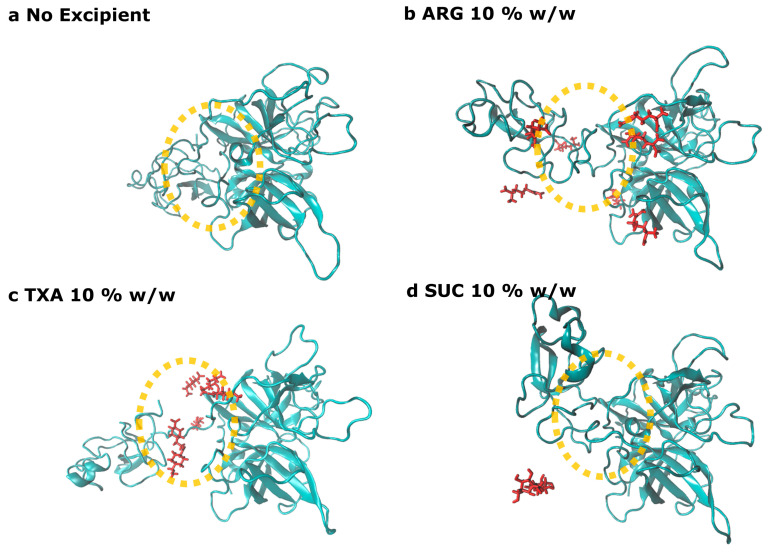
The domain separation between the kringle-2 and catalytic domains after the reconstitution. The representative structures are obtained from the final frame of the simulations. The structures are aligned on the catalytic domain. The following structures are shown: Reteplase with: (**a**) no excipient, (**b**) ARG 10% *w*/*w*, (**c**) TXA 10% *w*/*w*, and (**d**) SUC 10% *w*/*w*. The connecting loop between the two domains is highlighted with an orange circle. The excipient located close to the connecting loop is shown as red sticks.

The radius of gyration gradually decreased during the FD process (Figure 5), indicating that the removal of water caused a loss of the space-filling protein–water hydrogen bond networks and led to a shrinkage of the protein. The level of protein compactness and protein hydration was related. Visual inspection of the simulations revealed that the linker between the kringle-2 and the catalytic domains collapsed when the water molecules were removed. This led to a self-interaction between the two domains in the Reteplase structure that could still be present after reconstitution (Figure 7a). The protein shrinkage could be reduced by the presence of the excipient in a concentration-dependent manner. A complete domain separation could be obtained in the presence of ARG or TXA at 10 *w*/*w* (Figure 7b,c). The domain connecting loop region was negatively charged (Figure 3a) and, therefore, both ARG and TXA were able to interact with the connecting region and hence stabilize the distance between the kringle-2 and the catalytic domains. In contrast, SUC, as a preferentially excluded excipient, showed no strong affinity toward the protein and was not able to stabilize the gap between the two domains during the simulations (Figure 7d). 

Due to the absence of polar solvent molecules in the dried state, the hydrophobic residues were no longer forced to remain in the core of the protein. Theoretically, it was expected that the protein would be more hydrophobic during the drying process. The hydrophobicity was monitored by calculating the ratio between the non-polar and the total solvent-accessible surface area of the protein (Figure 6). The non-polar SASA ratio increased during the FD simulations, reflecting the exposure of hydrophobic residues. The increase in hydrophobicity could be prevented by adding excipients to the system. Better stabilization of the protein could be achieved at the higher concentration of the excipients (Figure 6). Interestingly, all three excipients could prevent the increase of hydrophobicity at the highest concentration. When it came to reversibility during reconstitution, the non-polar surface ratio showed a good recovery of the locally exposed hydrophobic residues, even without the presence of the excipients (Figure 6). In contrast, the radius of gyration could not be completely recovered by the addition of water, which indicated that it was more difficult to recover the change caused by the collapse of the global structure compared with the local exposure of the hydrophobic residues.

The freeze-drying process also affected the secondary structure. We recorded the β-sheet content as a function of simulation time (Figure S4). During the drying simulations, a slight decrease in β-sheet content could be observed independent of the presence of the excipients. However, the recovery of the β-sheet content during the reconstitution could only be observed in the presence of excipients.

The simulations revealed that some of the conformational changes that occurred during the FD simulation were reversible. When water was re-added to the system without excipients, both the radius of gyration and non-polar SASA showed some improvement compared with Reteplase at the 2D state. However, the presence of excipients showed a better recovery rate compared with simulations without any excipients (Figure 5 and Figure 6) during the reconstitution simulations. On the contrary, the domain collapse of Reteplase could be recovered only when ARG or TXA were present in the system (Figure 7). It is suggested that the reversibility of the FD-induced damage depends on the complexity of the processed protein (i.e., architecture/topology and single domain/multi-domains) and the presence of excipients to assist in the recovery of the conformational changes during the reconstitution phase. 

Conformational changes could be mainly observed during the drying and reconstitution simulations. During the freezing simulations, no conformational change could be observed. In contrast, Arsiccio et al. 2018 reported an immediate increase in the non-polar SASA ratio for human growth hormone during the first 20 ns of their freezing simulations [4]. In their study, no pre-simulations were performed before freezing and the simulations were compared between two cold (simulations without ice block) 100 ns simulations with two different water models: TIP4P and TIP4P/ICE. Since the model proteins, simulation setups, and simulation times were different, a direct comparison was not possible.

Duran and co-workers performed freezing MD simulations of lactate dehydrogenase [57]. The authors carried out separate RT and freezing simulations. Compared with RT simulations, the authors observed a decrease in root mean square fluctuation (RMSF), radius of gyration, and total SASA and an increase in intramolecular hydrogen bonds in the freezing simulations. A decrease in RMSF was always expected since the freezing conditions had lower temperatures. A slight decrease in total SASA and radius of gyration could also be observed during our freezing simulations. However, for some trajectories, we could observe an increase in the radius of gyration when the simulations entered the freezing stage (Figure 5a: ARG 10 mM; Figure 5b: TXA 100 mM). The time evolution of the RMSD (Figure 4) showed that the freezing simulations tended to preserve the protein structure, independent of the starting RMSD of the freezing condition. Note that the structural properties sampled during freezing simulations without any bias could be influenced by the initial conformations used to initiate the freezing simulations. In other words, the temperature and the sampling time in the freezing simulations were too low to sample major structural changes.

The average number of intramolecular hydrogen bonds within Reteplase during the last 200 ns of the simulations without excipients was calculated to be 66 for RT simulations and 70 for F simulations, which indicated a slight increase in protein compactness, which also reflected the decrease in the radius of gyration (Figure 5).

### 3.3. Local Structural Changes and Effects of Excipients

**Figure 8:**
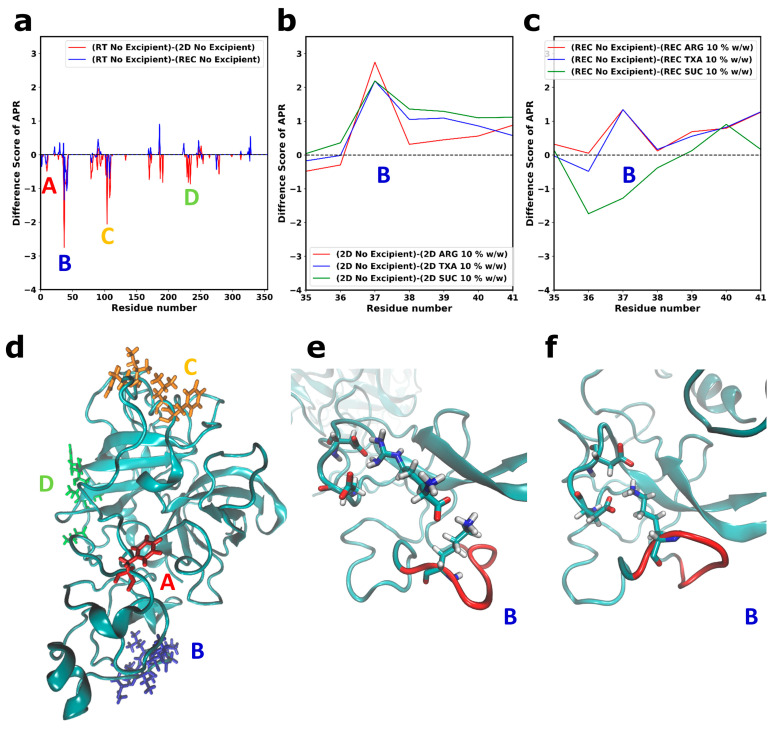
The difference scores between aggregation-prone residues (APR). The scores are calculated using A3D [56] and are based on taking the average of scores determined from the final frame of the two replicates. A negative score indicates that the residue becomes more aggregation prone. (**a**) The outputs from the RT simulations are compared to the secondary drying (2D) and reconstitution simulations (REC) without excipients. The local stabilizing effect of excipients (10% *w*/*w*) is monitored during: (**b**) 2D simulations and (**c**) REC simulations. The APRs that become more pronounced during the drying simulations are highlighted with colored letters (A–D). (**d**) The same APRs are highlighted in the Reteplase structure. (**e**,**f**) Effect of local excipient interactions. Lys40 in the kringle-2 region can interact with the carboxylic group of excipients. (**e**) Interaction between Lys40 and an ARG molecule is shown as an example. (**f**) Lys40 interacts with Asp64 and Asp66 in the presence of SUC. The snapshot is taken from the final frame of the reconstitution simulation with 10% *w*/*w* excipients. The local aggregation-prone region (Met35-Val41) is colored red.

To screen the local structural changes, we monitored the change in aggregation-prone residue (APR) scores of the final frame of the 2D and REC simulations using A3D. The CG simulations revealed that the aggregation-prone residues were directly involved in the PPIs (Figure 3b–d). Therefore, an increase in APRs may have increased PPIs and consequently affected protein stability. To determine the change in the APR scores between the FD processes, the APR scores of the RT simulations were subtracted from the APR scores from 2D or REC simulations; the results are presented in Figure 8. The APR score was typically used to determine the likelihood of a residue to contribute to protein aggregation, with a higher positive score indicating a higher propensity for aggregation. 

We calculated a difference score; a negative difference score indicated that the protein became more aggregation-prone compared with the RT simulations. The regions that became significantly more aggregation-prone during the drying simulations are mapped in Figure 8a as APR A, B, C, and D. The region that became more aggregation-prone during the drying simulations is shown in Figure 8d. We observed that the formation of hydrophobic clusters led to the local increase in the aggregation propensity score compared with the simulations at RT. Figure 8a shows that some aggregation-prone residues could be stabilized during reconstitution (APR C and D in Figure 8a), as indicated by a decrease in the difference in APR scores.

The obtained result was in accordance with the non-polar SASA ratio (Figure 6). The 2D simulations caused a relatively high increase in the hydrophobic protein surface. However, during reconstitution, the surface polarity almost reached its original state prior to FD. Without the presence of an excipient, the complete recovery of the surface polarity could not be achieved, as seen from both the non-polar SASA ratio (Figure 6) and the APR difference scores (Figure 8a).

We could observe that a certain APR could not be recovered after reconstitution (Figure 8a, APR B). To investigate whether the excipients caused a local stabilization in this region during 2D and REC, we calculated the difference between APR scores obtained from the no-excipient simulations and the APR scores obtained from the simulations with excipients. Results are shown in Figure 8b,c for, respectively, 2D and REC. A negative score indicated that the addition of excipients caused an increase in aggregation propensity and vice versa.

A stabilization of APR B scores could be observed during the secondary drying when the excipients were added to the system (Figure 8b and Figure S8). The decrease in the aggregation propensity was caused by the interactions between protein and excipients. The presence of ARG or TXA improved the local stability of Reteplase during the reconstitution phase by stabilizing APR B (Figure 8c). However, SUC could not stabilize some local aggregation-prone regions of Reteplase. No stabilization could be observed at APR B in the kringle-2 domain (Figure 8c) that was located between Met35-Val41 (Figure 8a). This region was previously studied by Serrano et al. [16] using molecular docking and mutagenesis. The author suggested that Lys40 provided a binding site for the carboxylic group of TXA [16], which was in line with our simulation results. An example of the Lys40-carboxylic acid interaction (here ARG) is illustrated in Figure 8e, showing that the carboxylic group of excipients could interact with Lys40. The Lys40-ARG interaction subsequently induced a small local conformational change in the kringle-2 domain, where the intramolecular interactions between Lys40 and Asp residues (Figure 8f) were interrupted and consequently caused a reduction in the surface exposed APR B: Met35-Val41 (Figure 8c). The APR B was stabilized by the binding of the ARG molecule in this region. The interactions were dominant throughout the simulations and, during the last 200 ns of RT (REC) simulations, Lys40 interacted with ARG for 90% (87%) and with TXA for 62.5% (66%) of the simulation time. 

The all-atom MD simulations revealed two protein-specific stabilization mechanisms for Reteplase: (1) the separation between the kringle-2 and catalytic domain and (2) interactions with the specific excipient binding site Lys40. SUC, a universal stabilizer, could not provide this protein-specific stabilization. In addition, the investigation revealed that some conformational alterations that occurred during the FD simulations could be reversed during the reconstitution. The results demonstrated the complexity of designing excipient formulations. SUC could act as a good protectant during freeze drying; however, the presence of excipients with charged functional groups was required to completely stabilize Reteplase.

### 3.4. Experimental Validation

**Figure 9:**
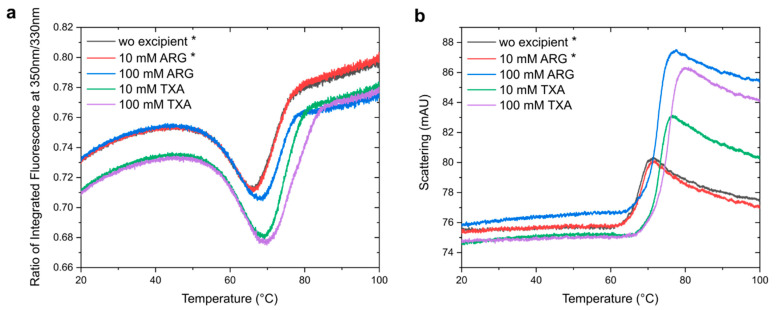
Effect of ARG and TXA in low and high concentrations on the thermal stability of Reteplase. (**a**) Thermal unfolding profiles of Reteplase with and without excipients measured with nanoDSF. (**b**) Aggregation during heating measured with the backscattering detector in nanoDSF. All curves are mean of triplicates. Due to aggregation after sample preparation, the protein concentration in the conditions marked with (*) was lower.

To experimentally verify our results, we used nanoDSF to investigate whether ARG and TXA had an influence on the thermal unfolding and aggregation onset temperatures (*T*_agg_) of Reteplase (Figure 9). The shape and cooperativity of the unfolding curves were not affected by the presence of ARG or TXA (Figure 9a). Since Reteplase already showed aggregation after sample preparation in the conditions without excipient and with 10 mM ARG, the protein concentrations for the nanoDSF measurements were slightly lower (marked with *). This could explain the different start and end ratios of the measurements for these two samples and could influence the melting temperatures (*T*_m_) and *T*_agg_ values. The *T*_m_ without excipient was 71.80 ± 0.03 °C, whereas *T*_m_ were 72.07 ± 0.05 °C and 73.44 ± 0.03 °C in the presence of 10 and 100 mM ARG, respectively. The TXA-containing samples had *T*_m_ of 74.78 ± 0.02 °C (10 mM) and 76.2 ± 0.1 °C (100 mM). The light scattering traces from nanoDSF revealed the distinct onset of aggregation for all conditions, following the same trend as the *T*_m_ values. Reteplase without excipients and 10 mM ARG showed a comparable aggregation onset of 62.6 ± 0.6 °C and 62.9 ± 0.4 °C, respectively. A shift to a higher *T*_agg_ was observed for 100 mM ARG (65.1 ± 0.5 °C) and TXA-containing samples, with *T*_agg_ values of 67.1 ± 0.6 (10 mM) and 67.4 ± 0.7 °C (100 mM). Interestingly, the high concentration conditions of both excipients shifted the onset of aggregation to higher temperatures but showed a larger increase in scattering signal, indicating larger or more aggregates. Nevertheless, 100 mM ARG and TXA in low and high concentrations significantly improved the thermal stability of Reteplase. These findings agreed with the literature findings as described above and supported our CG simulations with and without ARG. 

Since it was experimentally not possible to detect conformational changes and excipient binding during the freezing and drying stages, we freeze thawed (FT) and lyophilized/ reconstituted (LYO) the Reteplase samples with and without excipients, ARG, and TXA to achieve an estimate of the protein stability during these stresses and furthermore understand whether the conformational changes were reversible. The samples were analyzed with high-performance size exclusion chromatography (HP-SEC) and CD spectroscopy prior to and after FT and LYO. 

**Figure 10:**
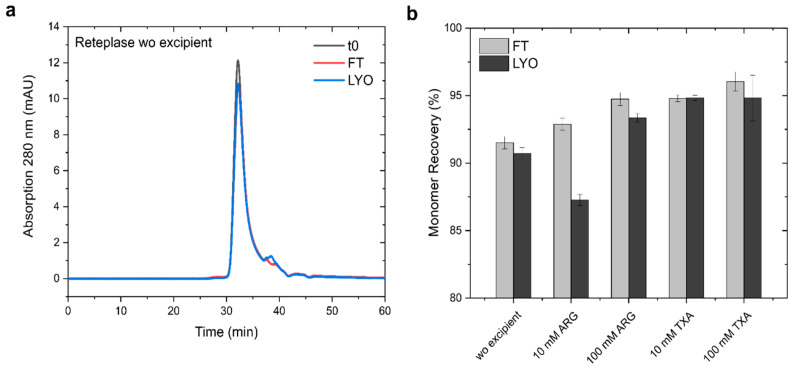
Freeze-thaw and lyophilization stress-induced aggregation of Reteplase. (**a**) SEC chromatograms of Reteplase without excipient before (t0) and after FT and LYO. (**b**) Monomer recovery (%) of Reteplase detected by SEC. Time point t0 was set to 100% for each condition.

The SEC chromatograms of Reteplase monitored at 280 nm showed a distinct monomer peak at ~32 min elution time and a second small peak at ~38 min elution time, indicating minor fragmentation of the protein (Figure 10). After FT and LYO, the monomer peak decreased, whereas the second peak only marginally increased, suggesting that Reteplase formed larger aggregates that were removed before injection onto the SEC column. To better visualize the monomer loss and to compare between the different excipients and concentrations, we calculated the monomer recovery from the peak area of the SEC chromatograms. The peak area from the unstressed sample was set as the 100% reference for each condition. We could observe monomer loss for all conditions, ranging from ~4% to ~8.5% during freeze thawing and ~5% to 13% after lyophilization and reconstitution. Both excipients had a stabilizing effect during both FT and LYO stress. However, ARG only seemed to prevent monomer loss at higher concentrations during LYO. Interestingly, TXA did not show a significant concentration dependency. The effects of ARG and TXA were in accordance with our nanoDSF measurements and showed a similar trend to our freeze-drying simulations. 

**Figure 11:**
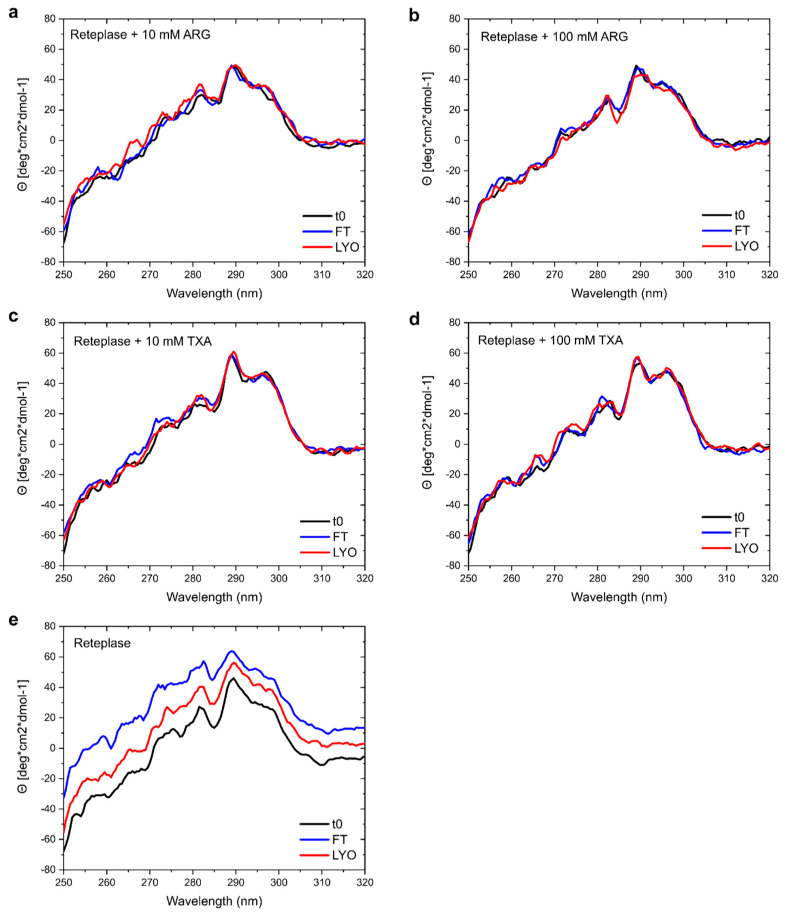
Effect of FT- and LYO-stress on Reteplase in the absence and presence of ARG and TXA measured by near-UV CD. (**a**) Spectrum of Reteplase with 10 mM ARG, (**b**) with 100 mM ARG, (**c**) with 10 mM TXA, (**d**) with 100 mM TXA, and (**e**) without an excipient.

To further evaluate whether we could verify the stabilization of Reteplase observed in our MD simulations, we analyzed the samples prior to and after FT-/LYO-stress with near-UV CD (Figure 11). The spectra of the protein with ARG (Figure 11a,b) and TXA (Figure 11c,d) at both concentrations before and after stress were superimposable with only minor deviations, indicating that the excipients prevented damage to the protein tertiary structure during freezing and freeze drying. Surprisingly, the CD spectra of Reteplase without excipients were shifted in Θ, even though the shape of the spectra remained comparable. The shift may have been caused by aggregates in the samples. Although the CD spectra of Reteplase with different excipients remained the same, conformational changes could occur, since near-UV CD monitored the change in the environment of aromatic residues. Hence, conformational changes that did not involve environmental changes of aromatic residues could occur. 

A study combining both simulations and experiments provided an atomic-level understanding of the complex behavior of biomolecular systems [58]. The experimental data showed both the weaknesses and strengths of the MD simulations. In experiments, the obtained signal was highly affected by the aggregation of Reteplase. Running a freeze-drying-induced aggregation simulation was far from a trivial task. However, the MD simulation could model the configuration change of Reteplase monomer, which was extremely difficult to study in experiments due to the protein aggregation. Considering that FD-induced partial unfolding that might lead to denaturation would increase the exposure of aggregation-prone residues of Reteplase (Figure 8), it was suggested that the denaturation of Reteplase was a contributing factor to the aggregation process of Reteplase. Using CG simulations, we were able to determine residues involved in monomer–monomer interactions. However, the results were only of qualitative nature due to the limitation of atomic resolutions and simulation scales of the current CG scheme. The simulation sampling could be improved by running more replicates and larger systems. However, increasing system size would inevitably require significantly more computational resources; therefore, accurate sampling of PPI similar to realistic aggregation behavior would be highly challenging. It should also be noted that each step of the FD simulation step was limited to 400 ns. Considering that the FD process can take day(s) to execute [59], the slow-mode changes during the FD process were not trivial to simulate. Therefore, even though the experimental data contained high noise levels due to the aggregation process, the experimental data were necessary to access the macroscopic phenomena that occurred during the FD process, which was extremely challenging to simulate. 

## 4. Conclusions

The FD simulations of Reteplase have shown different protein-specific and protein-unspecific effects of excipients that can occur during the freeze-drying and reconstitution processes. The protein-unspecific effects during freeze drying are manifested in the decrease of RMSF and radius of gyration, which are accompanied by an increase in hydrophobicity. The mobility of the protein will always decrease due to the low temperature during freezing and the primary drying process. During dying, a slight reduction of the radius of gyration due to the loss of space-filling water molecules can be observed. In addition, the loss of water molecules during the drying simulations causes the protein to shrink and the hydrophobic protein surface to increase. These conformational changes can be reduced by adding lyoprotectants independent of the protein structure.

However, the structural complexity of the protein will affect the reversibility of the damage that is caused by the freeze-drying process. The decreased radius of gyration and increased hydrophobicity can be recovered during reconstitution. In contrast, a collapse of a multi-domain structure is irreversible. The result suggests that specific binding must be mediated through charged functional groups to stabilize the domain separation of Reteplase. Interestingly, the domain separation of Reteplase cannot be achieved by adding SUC to the system. In addition, Lys40 in the kringle-2 domain provides a binding site for the negatively charged functional group of excipients. A salt bridge between Lys40 and ARG or TXA causes a local decrease in the aggregation propensity. Finally, the PPIs can also be altered by the addition of ARG. The CG simulations suggest that the solubility of Reteplase can be significantly increased in the presence of ARG by decreasing PPIs.

The study reveals challenges in developing a stabilizing formulation for the FD process, where some proteins require a specific type of excipient to achieve a stable formulation.

## Figures and Tables

**Table 1:** Overview of the FD simulations. The Reteplase-containing systems were simulated in duplicates with different excipient settings.

Excipient	Concentration	Number of Excipient Molecules	Number of Ions
No Excipient	No Excipient	No Excipient	1 Na^+^
ARG	10% *w*/*w*	284	283 Cl^−^
ARG	100 mM	51	50 Cl^−^
ARG	10 mM	5	4 Cl^−^
TXA	10% *w*/*w*	314	1 Na^+^
TXA	100 mM	51	1 Na^+^
TXA	10 mM	5	1 Na^+^
SUC	10% *w*/*w*	144	1 Na^+^

## Data Availability

Not applicable.

## Supplementary Materials

The following supporting information can be downloaded at: https://www.mdpi.com/article/10.3390/pharmaceutics15071854/s1, Figure S1: Protein–protein interaction heatmap of Reteplase with arginine 10% *w*/*w*; Figure S2: Protein–protein interaction heatmap of Reteplase without any excipient; Figure S3: Solubility (i.e., oligomerization) of Reteplase; Figure S4: Time evolution of Reteplase’s RMSD calculated with respect to the initial simulation structure for (a) Reteplase with ARG, (b) Reteplase with TXA, and (c) Reteplase with SUC at different concentrations; Figure S5: Time evolution of RMSD for Reteplase and its corresponding domains; Figure S6: The time course of the β-sheet contents of Reteplase without and with the addition of different excipients; Figure S7: The time course of the RMSD of Reteplase+ SUC 10 *w*/*w*. (a) The complete RMSD time course. The reference frame is the initial structure of the RT simulation. (b) The RMSD time course of only the FD + reconstitution processes. The reference frame is the initial structure of the F simulation; Figure S8: The predicted aggregation propensity score of the Reteplase residues for 2D simulations without and with the addition of (a) ARG 10% *w*/*w*, (c) TXA 10% *w*/*w*, and (e) SUC 10% *w*/*w* and REC simulations without and with addition of (b) ARG 10% *w*/*w*, (d) TXA 10% *w*/*w*, and (f) SUC 10% *w*/*w*.
