# Supplementary material for: Combining Molecular Dynamics Simulations and Biophysical Characterization to Investigate Protein-Specific Excipient Effects on Reteplase during Freeze Drying
Source: Pharmaceutics. 2023 Jun 30;15(7):1854. doi: 10.3390/pharmaceutics15071854 (PMC10384596; doi:10.3390/pharmaceutics15071854)
Supplement: Supplementary file 1 [file pharmaceutics-15-01854-s001.zip › pharmaceutics-2430318-supplementary.docx]

Supplementary Materials

Combining Molecular Dynamics Simulations and Biophysical
Characterization to Investigate Protein-Specific Excipient
Effects on Reteplase during Freeze Drying

Suk Kyu Ko, Gabriella Björkengren, Carolin Berner, Gerhard Winter, Pernille Harris and Günther H. J. Peters

Supplement A: Supplementary Results for the Aggregation Study of Reteplase

Reteplase becomes much more soluble in the presence of arginine (ARG) at 10 % *w*/*w*. The protein-protein interaction heatmaps are displayed in Figure S1 (heatmap in the presence of ARG) and Figure S2 (heatmap without any excipient). The visual representation of the solubility of Reteplase during the coarse-grained (CG) simulations is shown in Figure S3.


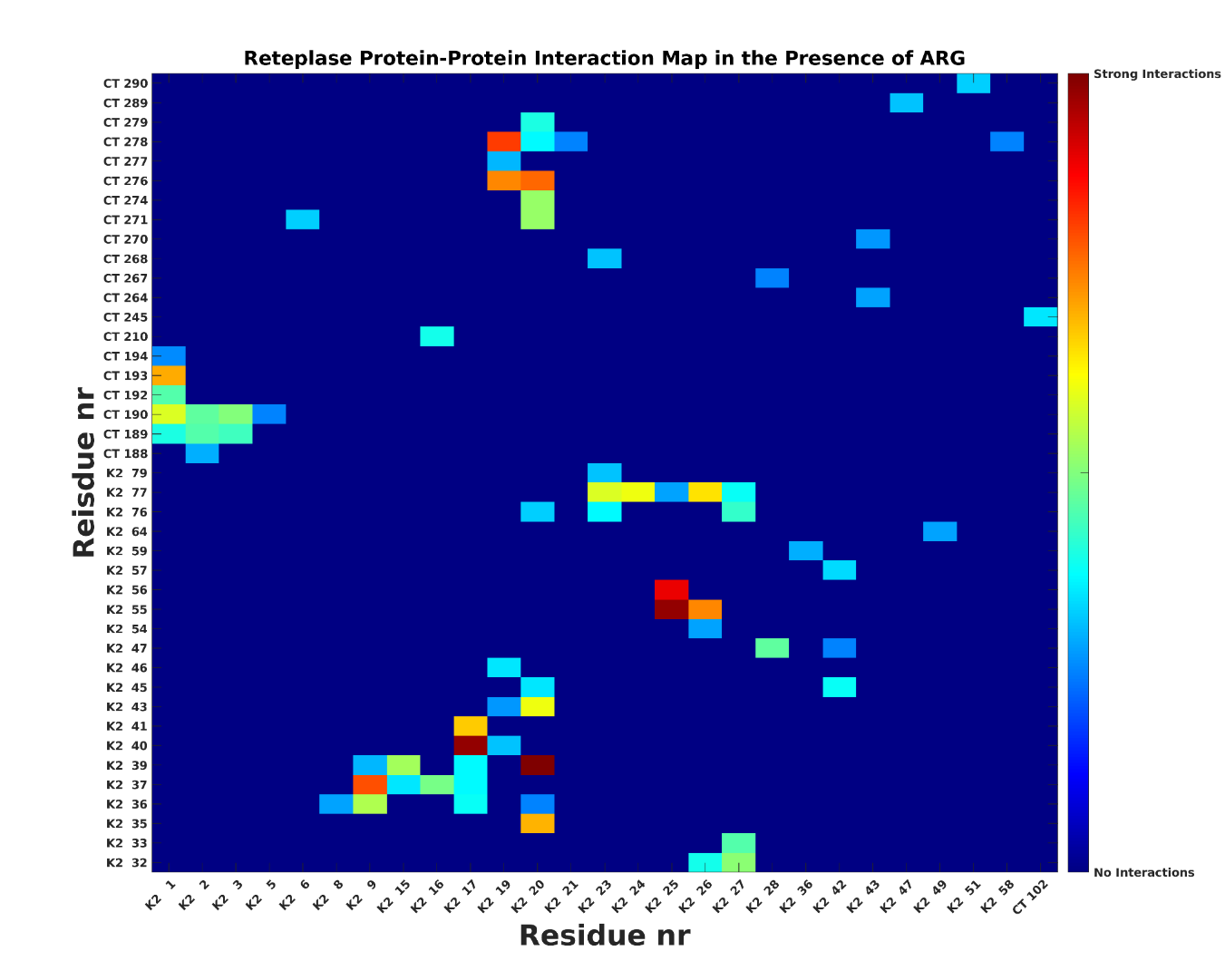


**Figure S1.** Protein-protein interaction heatmap of Reteplase with ARG 10 % w/w. The residue number of the interacting residue pair is shown in the x- and y-axis of the heatmap. The heatmap is derived from the observed interactions from all three simulations. K2 and CT denote the kringle-2 and catalytic domains, respectively. The color scheme of the heatmap is scaled to the strongest interaction that could be observed from the CG simulations of Reteplase + ARG.


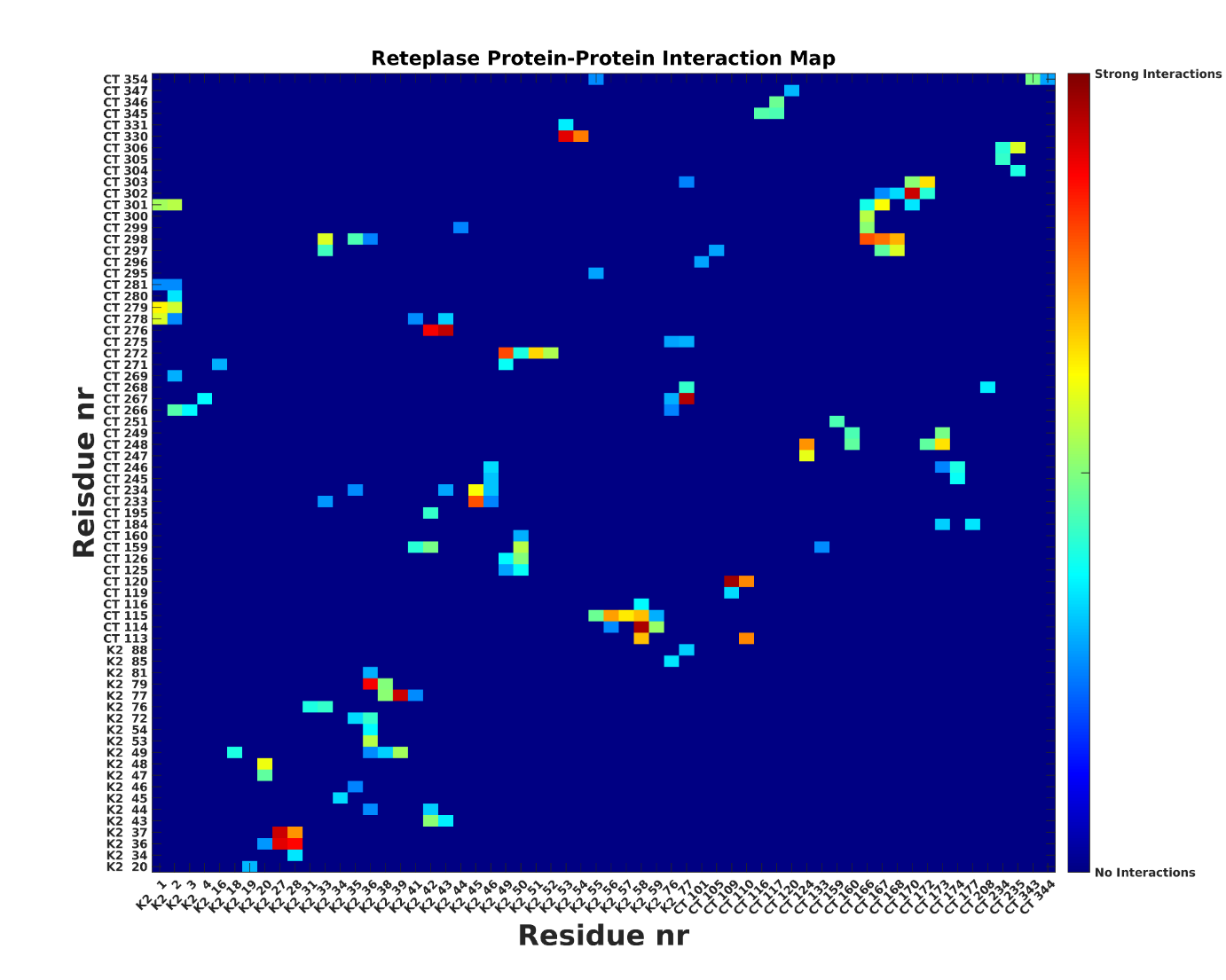


**Figure S2.** Protein-protein interaction heatmap of Reteplase without any excipient. The residue number of the interacting residue pair is shown in the x- and y-axis of the heatmap. The heatmap is derived from the observed interactions from all three simulations. K2 and CT denote the kringle-2 and catalytic domains, respectively. The color scheme of the heatmap is scaled to the strongest interaction that could be observed from the CG simulations of Reteplase + ARG.


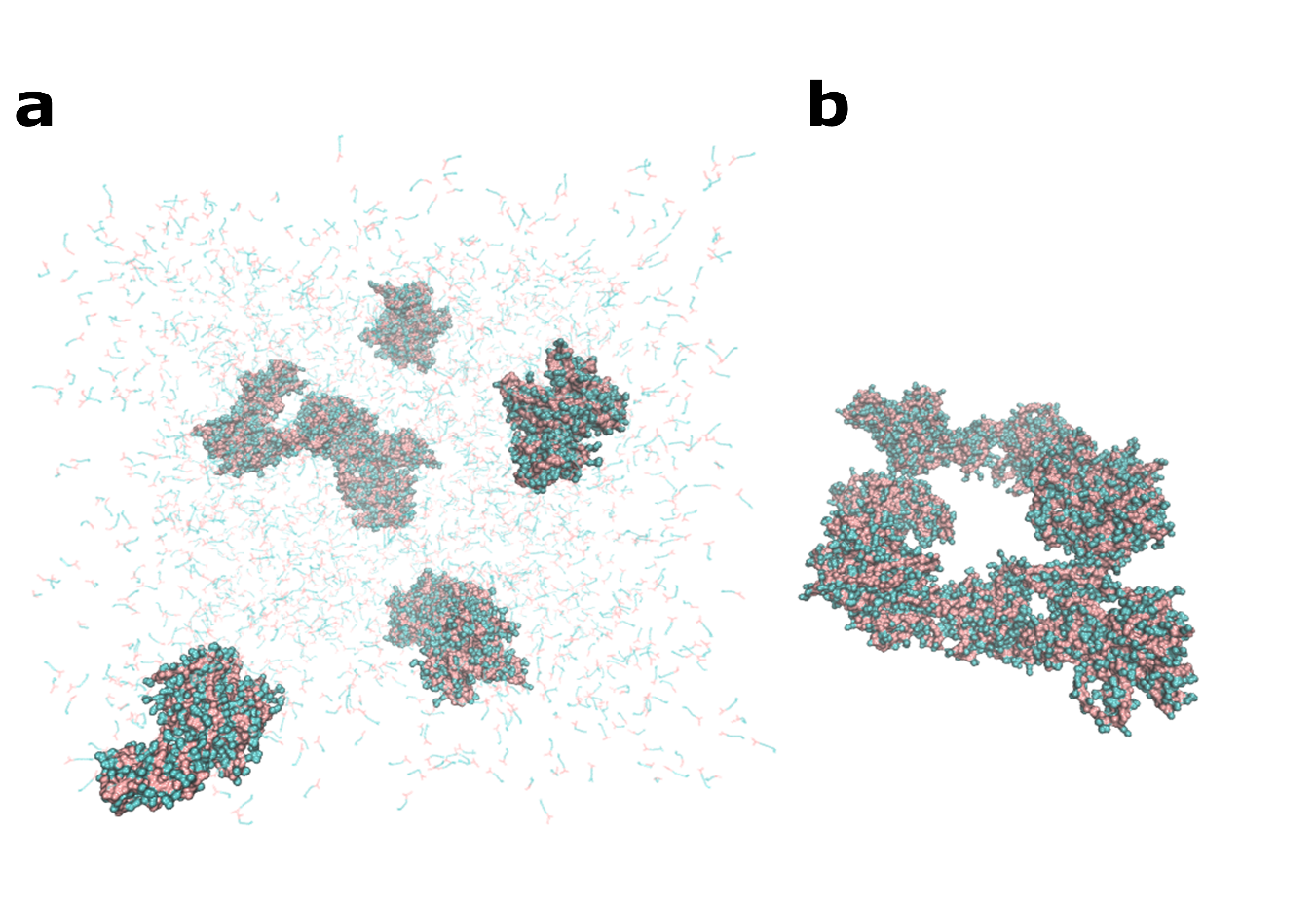


**Figure S3.** Solubility of Reteplase. The final frame from the CG simulations is shown for the systems: (**a**) with ARG 10 % *w*/*w*, (**b**) without arginine. The Reteplase monomers are shown in the bead-like representations. The ARG excipient is shown in sticks.

Supplement B: Supplementary Results of the Freeze-Drying Simulations of Reteplase

The complete root mean square deviation (RMSD) plot of the FD simulations is shown in Figure S4, following excipients were considered: ARG, tranexamic acid (TXA), and sucrose (SUC). The RMSD plots of kringle-2 and catalytic domains are shown in Figure S5. Figure S6 shows the time course of the beta-sheet contents of Reteplase with the different excipients during the freeze-drying simulations. Figure S7 compares two RMSD plots of Reteplase + SUC 10 % *w*/*w*: (a) the complete RMSD time course, and (b) the RMSD time course of only the FD + reconstitution processes. The aggregation propensity score of Retepase during the secondary drying (2D) and reconstitution (REC) simulations is shown in Figure S8.


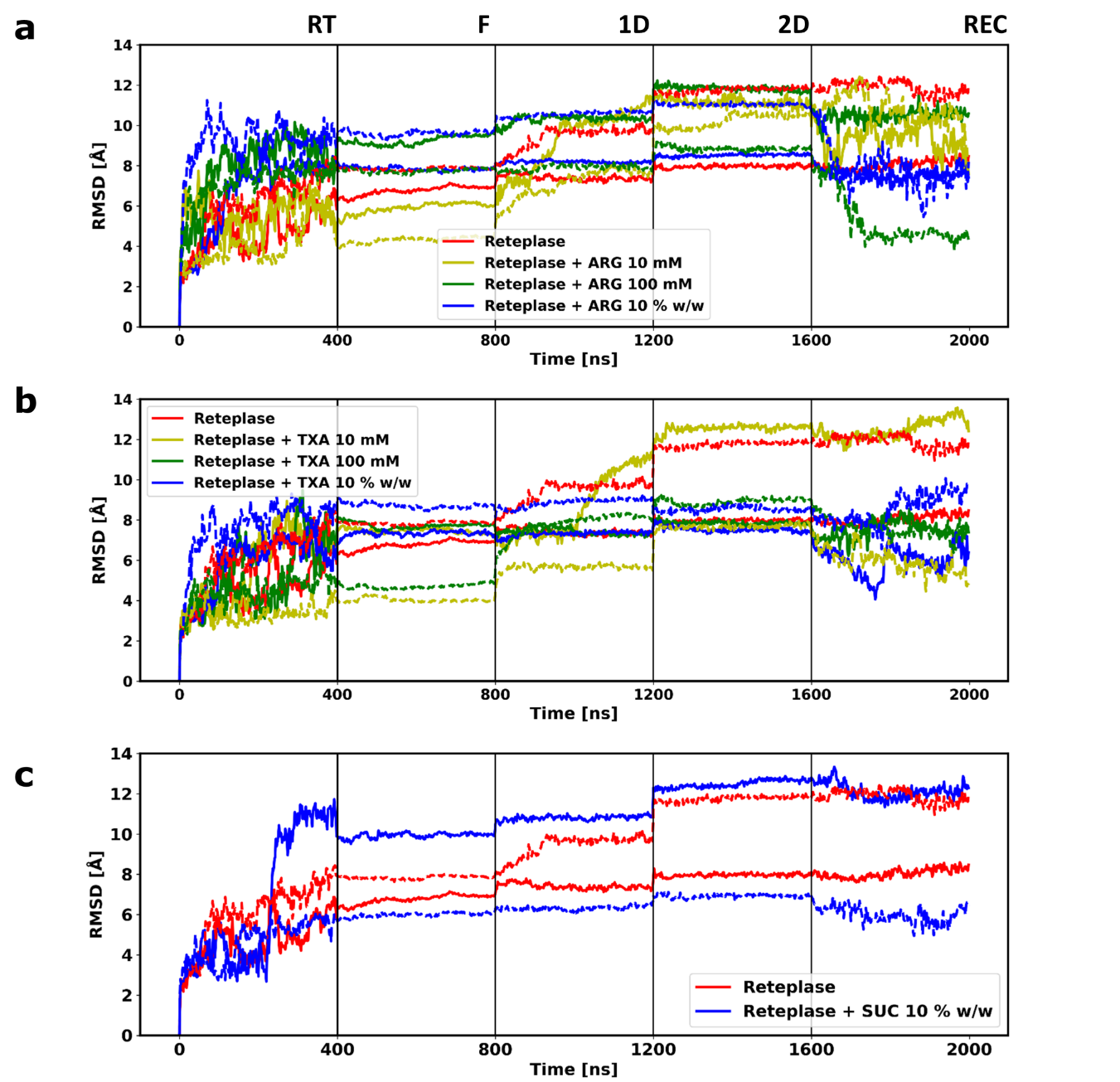


**Figure S4.** Reteplase RMSD plots calculated with respect to the initial simulation structure. 3 subplots display the RMSD of the system with different excipients at different concentrations: (**a**) Reteplase with ARG, (**b**) Reteplase with TXA, and (**c**) Reteplase with SUC. The replicate simulations are represented with dotted lines. The vertical lines separate the different stages in the FD simulations: room temperature (RT), freezing (F), primary drying (1D), secondary drying (2D), and reconstitution (REC).


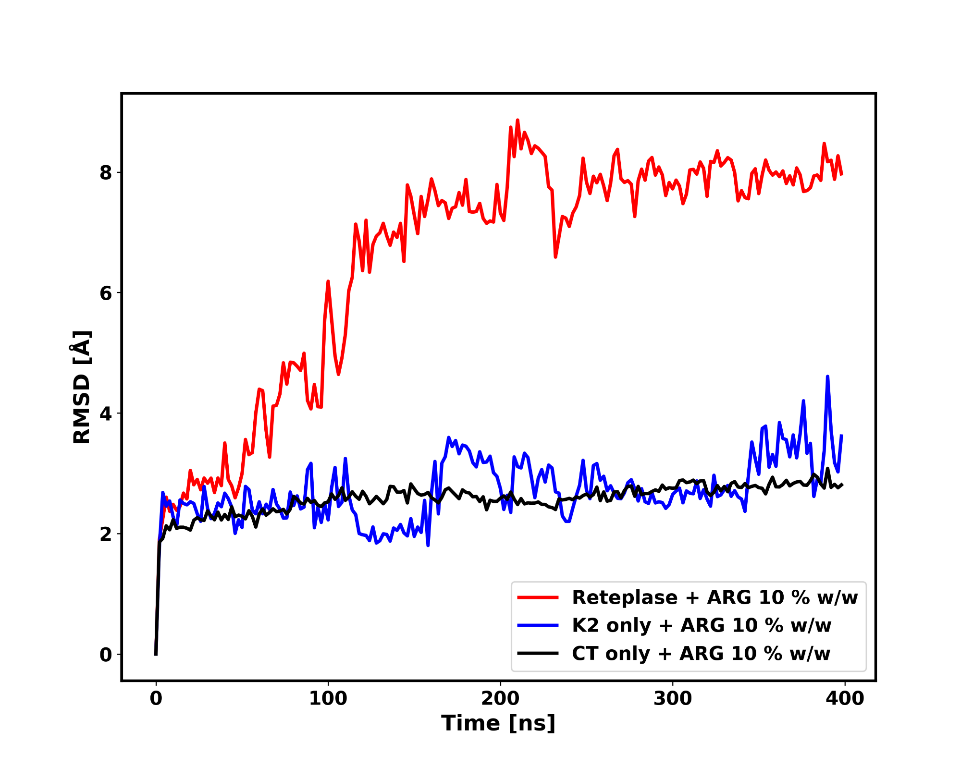


**Figure S5.** RMSD of Reteplase divided into separate domains. The magnitude of RMSD increase during the room temperature can be significantly reduced by plotting the kringle-2 and catalytic domains in a separate time course. K2 and CT denote the kringle-2 and catalytic domains, respectively.


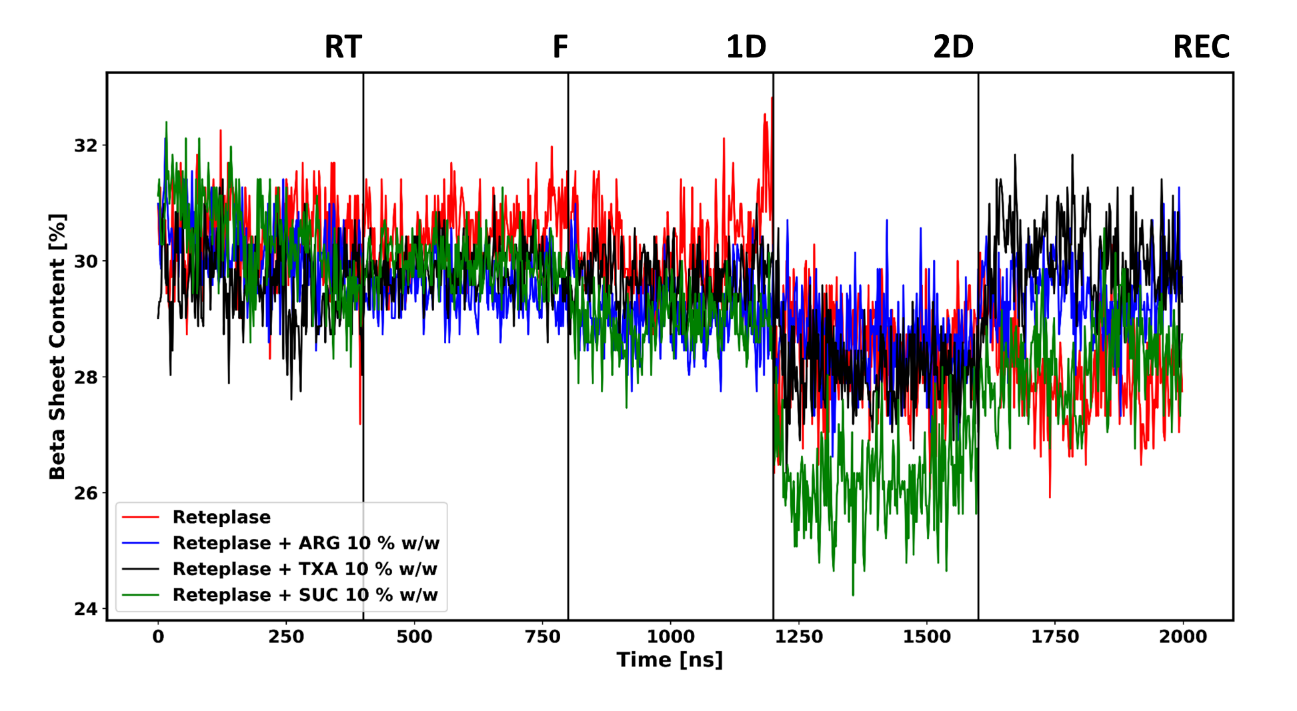


**Figure S6.** The time course of the β-sheet contents of Reteplase with the presence of different excipients. Each time course is obtained from the average of the two trajectories. The vertical lines separate the different stages in the FD simulations: room temperature (RT), freezing (F), primary drying (1D), secondary drying (2D), and reconstitution (REC).


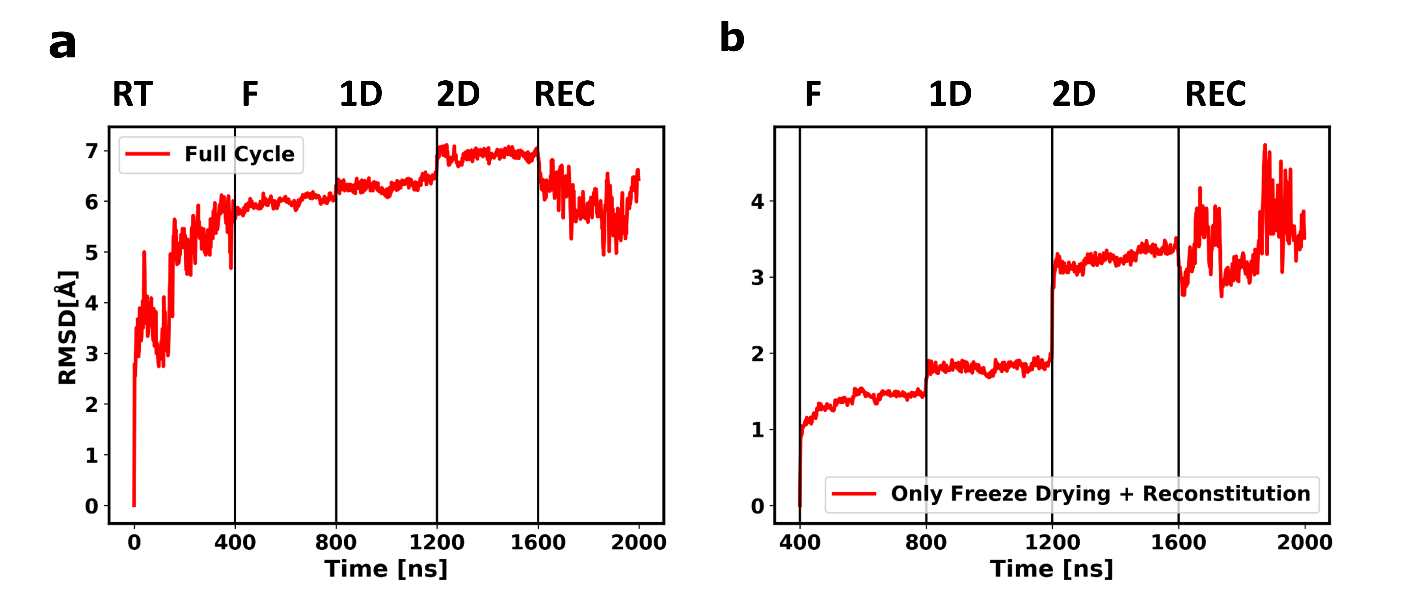


**Figure S7.** Representative RMSD *vs*. time plot of Reteplase + SUC 10 % *w*/*w* during the FD simulations. Room temperature (RT), freezing (F), primary drying (1D), secondary drying (2D), and reconstitution (REC). (**a**) The complete RMSD time course. The reference frame is the initial structure of the RT simulation. (**b**) the RMSD time course of only the FD + reconstitution processes. The reference frame is the initial structure of the F simulation.


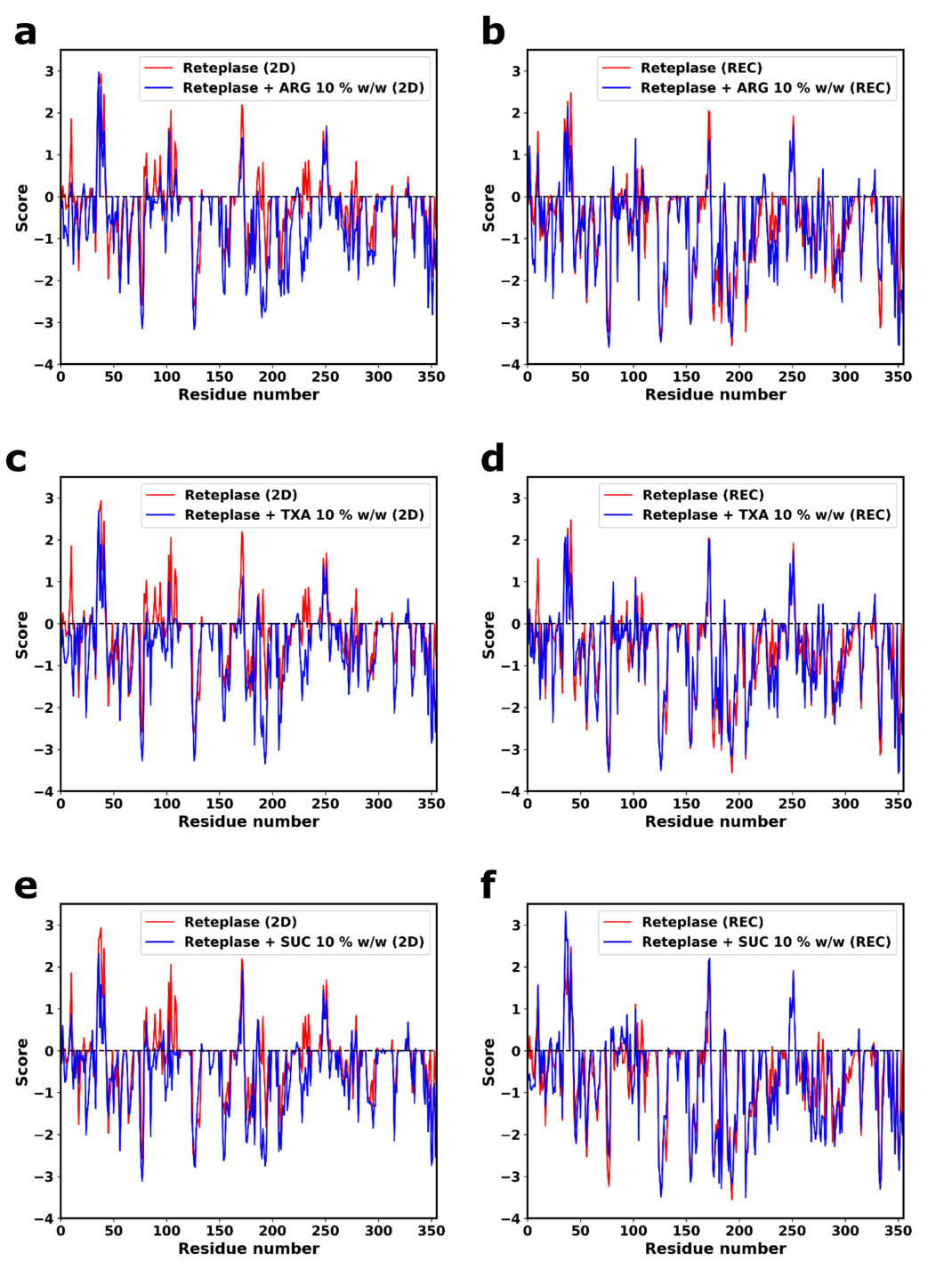


**Figure S8.** The predicted aggregation propensity score of the Reteplase residues. The score was calculated using Aggrescan3d. A positive score indicates that the residue is prone to aggregate. The soluble residues are scored with a negative value. The buried residues have a zero score. The score is obtained from the average of the final frame of the secondary drying simulations (left), and reconstitution simulations (right). The red score indicates Reteplase without excipients. The blue score is obtained from Reteplase with: a)-b) ARG 10 % *w*/*w*, c)-d) TXA 10 % *w*/*w*, and e)-f) SUC 10 % *w*/*w*.
